# Periodontitis impacts on thrombotic diseases: from clinical aspect to future therapeutic approaches

**DOI:** 10.1038/s41368-024-00325-9

**Published:** 2024-10-15

**Authors:** Jinting Ge, Xuanzhi Zhu, Chengxin Weng, Ding Yuan, Jichun Zhao, Lei Zhao, Tiehao Wang, Yafei Wu

**Affiliations:** 1 Division of Vascular Surgery, Department of General Surgery, West China Hospital, Sichuan University, Chengdu, China; 2 State Key Laboratory of Oral Diseases & National Center for Stomatology & National Clinical Research Center for Oral Diseases & West China Hospital of Stomatology, Sichuan University, Chengdu, China; 3 Department of Periodontics, West China Hospital of Stomatology, Sichuan University, Chengdu, China

**Keywords:** Periodontitis, Cardiovascular diseases

## Abstract

Periodontitis is a chronic inflammatory disease initiated by biofilm microorganisms and mediated by host immune imbalance. Uncontrolled periodontal infections are the leading cause of tooth loss in adults. Thrombotic diseases can lead to partial or complete obstruction of blood flow in the circulatory system, manifesting as organ or tissue ischemia and necrosis in patients with arterial thrombosis, and local edema, pain and circulatory instability in patients with venous thrombosis, which may lead to mortality or fatality in severe case. Recent studies found that periodontitis might enhance thrombosis through bacterial transmission or systemic inflammation by affecting platelet-immune cell interactions, as well as the coagulation, and periodontal therapy could have a prophylactic effect on patients with thrombotic diseases. In this review, we summarized clinical findings on the association between periodontitis and thrombotic diseases and discussed several novel prothrombotic periodontitis-related agents, and presented a perspective to emphasize the necessity of oral health management for people at high risk of thrombosis.

## Introduction

Periodontitis is a chronic inflammatory disease initiated by biofilm microorganisms and mediated by host immune imbalance,^1^ which is characterized by the destruction of tooth-supporting tissues, including the gingiva, alveolar bone, periodontal ligament, and cementum,^2^ and it is the leading cause of tooth loss in adults.^3–5^ The overall prevalence of periodontitis is as high as 45%-50% globally, with the most severe cases affecting 11.2% of the world’s population, ranking it the sixth most prevalent disease in humans.^6^ Extensive evidence suggests a strong association between periodontitis and various systemic diseases such as atherosclerosis,^7^ rheumatoid arthritis,^8^ inflammatory bowel disease,^9^ diabetes mellitus (DM),^10–12^ and chronic obstructive pulmonary disease.^13^ The keystone pathogens in periodontitis, represented by *Porphyromonas gingivalis* (*P. gingivalis*), can invade the blood circulation directly through ulcers in the periodontal pockets and colonize distal organs to produce toxins that trigger inflammation.^14^ Periodontal pathogen can also invade the intestinal mucosal barrier via the oral-gut axis, causing gut dysbiosis.^15^ In addition, the release of damage-associated molecular patterns (DAMPs) due to localized periodontal tissue destruction and subsequent systemic immune responses also contribute to an increased inflammatory burden in other organs.^16^ The high prevalence of periodontitis worldwide necessitates the management of oral cavity infection and inflammation as an inevitable health concern for patients with systemic diseases. Therefore, it is crucial to focus on prevention or treatment of periodontitis in order to effectively control this risk factor. The association between periodontitis and cardiovascular disease (CVD) has been extensively investigated over the last three decades. According to World Health Organization (WHO), CVD are a group of disorders of the heart and blood vessels, which include coronary heart disease, cerebrovascular disease, peripheral arterial disease, rheumatic heart disease, congenital heart disease, deep vein thrombosis and pulmonary embolism and other conditions.^17^ CVD currently stands as the primary cause of mortality from non-communicable diseases worldwide. In 2022, CVD is projected to account for approximately 19.8 million global deaths, resulting in a loss of 396 million years of life as well as 44.9 million years lost due to disability (YLD).^18,19^ Notably, about one-third (34%) of cardiovascular-related deaths occur before the age of 70.^20^ Patients with severe periodontitis exhibit significant subclinical cardiovascular disease features when compared to their counterparts, such as impaired flow-mediated dilatation (FMD), severer arterial stiffness and calcification and markedly increased carotid intima-media thickness (cIMT).^21,22^ Epidemiologic studies have consistently demonstrated a heightened prevalence of coronary arterial disease,^23^ cerebrovascular disease,^24^ and peripheral arterial disease (PAD)^25^ among individuals affected by periodontitis. Patients with periodontitis are also at a higher risk of experiencing acute cardiovascular events, such as myocardial infarction (MI)^23^ and stroke.^24^ Furthermore, a large sample Asian study based on a health insurance database revealed that individuals with periodontitis exhibited a higher incidence of atrial fibrillation than those without periodontal disease and thus had a higher risk of arterial embolism events, which indicated that more attention should be paid to the risk of thrombotic diseases in patients with periodontitis.^26^

**Fig. 1:**
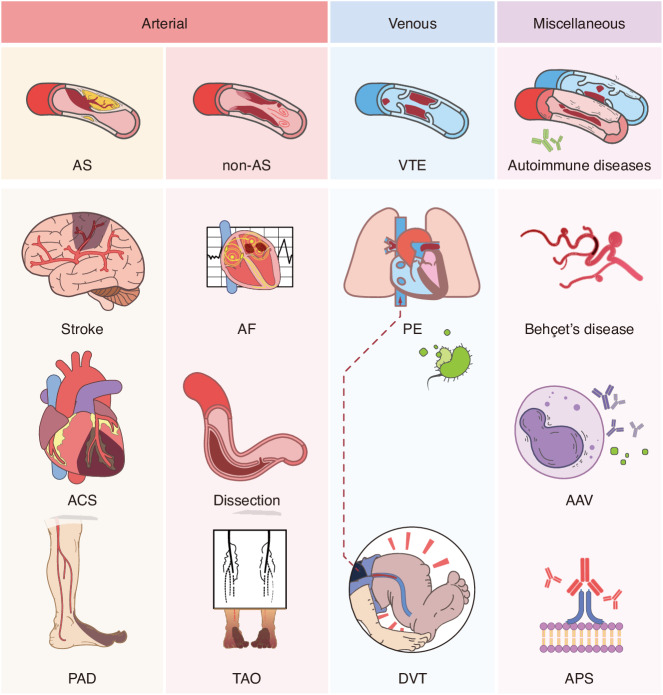
Representative periodontitis-related thrombotic diseases encountered in clinical practice. Arterial thrombotic diseases can be categorized as AS-related and nonAS-related, characterized by organ or tissue ischemia and necrosis. Stroke, acute coronary syndrome (ACS) and peripheral artery disease (PAD) are most common AS-related arterial thrombotic diseases, while thromboangiitis obliterans (TAO), atrial fibrillation (AF) and arterial dissection-related thrombosis are the most prevalent nonAS-related thrombotic diseases. Venous thromboembolism (VTE) encompasses deep vein thrombosis (DVT) and pulmonary embolism (PE), manifesting as edema, pain and circulatory instability. Autoimmune-related thrombotic disorders are relatively rare and can affect small vessels in both arterial and venous systems. Examples of such conditions include Behçet’s syndrome, anti-neutrophil cytoplasmic antibody (ANCA)-associated vasculitis (AAV) and antiphospholipid syndrome (APS). The illustration was created by C.W

Thrombotic diseases, which can be divided into arterial thrombosis (AT) and venous thrombosis (VT), constitutes a significant component within the realm of CVD^17^ and pose a significant global health and economic burden (Fig. 1). Thrombosis is a major and most feared complication of CVD, which plays a vital role in the pathophysiology mechanism of various CVD, including ischemic heart disease, ischemic stroke, and venous thromboembolism (VTE).^27–30^ Meanwhile, CVD patients have an increased risk of arterial thrombosis.^31^ Furthermore, arterial thrombosis and VTE can be directly activated when triggered by one or multiple factors in Virchow’s triad (sluggish blood flow, blood hypercoagulability, and endothelial dysfunction).^32^ Although the relationship between well-established risk factors such as diabetes mellitus, smoking, hypertension, and dyslipidemia and thrombosis is widely recognized, there are still certain thrombotic diseases whose mechanisms and etiology are difficult to explain by traditional risk factors.^33–35^ In recent years, considerable academic attention has been devoted to exploring the potential association between inflammation and thrombosis.^28,36^ This theory posits that systemic or localized vascular inflammation can lead to damage to the endothelial barrier, alterations in blood composition resulting in hypercoagulability, as well as excessive stress on immune cells involved in platelet activation.^28,37^ In atherosclerosis, the phenotypic transformation of macrophages and endothelial dysfunction driven by inflammation associated with pathogen or damage are pivotal mechanisms for plaque enlargement and instability.^38^ Similar mechanisms may also play a crucial role in thrombosis triggered by subsequent plaque rupture.^39^ In addition, long-term chronic systemic inflammation combined with traditional risk factors also elevates the risk of venous thrombosis,^40^ hence emphasizing the potential benefits of reducing systemic inflammation for both prevention and prognosis of thrombotic events.^27^

Currently, extensive research has been conducted on the association between periodontitis and CVD. However, most existing studies primarily focus on elucidating the pathophysiological mechanisms linking periodontitis to atherosclerosis.^7,41^ Although certain investigations have identified abnormal thrombotic indicators level in patients with periodontitis, there is a dearth of research exploring how periodontitis promotes thrombosis and contributes to related pathological mechanisms.^42–44^ Thus, this review comprehensively outlines the association and underlying mechanisms linking periodontitis and thrombotic diseases, offering valuable insights into the management of periodontal and cardiovascular health.

## Periodontitis can be the promoting factor for thrombosis

The association between periodontitis and periodontal therapy with various types of cardiovascular disease had been well documented in the consensus report of the joint workshop conducted by the European Federation of Periodontology and the World Heart Federation.^7^ Notably, the report highlights that patients with periodontitis have significantly elevated concentrations of circulating thrombotic factors (e.g., fibrinogen) and heightened hemocyte reactivity compared with healthy individuals.^42,45^ This implies that, apart from atherosclerosis-related CVD, periodontitis may also contribute to the systemic progression of all forms of thrombotic diseases.

### Periodontitis and arterial thrombosis

#### Atherosclerosis-related thrombosis

Atherosclerosis (AS) are typically not directly associated with the formation of occlusive thrombi due to the absence of pro-thrombotic conditions on the plaque surface.^27,46^ However, AS-related thrombosis can be triggered by the narrowing of arteries caused by atherosclerosis and the release of lipid cores from ruptured plaques.^27^ Although the role of periodontitis in the progression of AS plaque formation is well established,^47,48^ the association of periodontitis with thrombosis after plaque rupture and intravascular coagulation induced by endothelial injury or lumen narrowing remains incompletely elucidated.

Ischemic stroke develops mainly as a consequence of AS and thrombosis of the arteries supplying blood to the brain, resulting in narrowing or even complete occlusion of the vessel lumen and leading to focal acute insufficiency of cerebral blood supply. AS-related thrombotic cerebral infarction accounts for 40% to 60% of all cases.^49,50^ A systematic review concluded an elevated risk of initial cerebrovascular events in patients with clinically diagnosed periodontitis or more severe periodontitis.^23^ Sen et al. demonstrated an association between periodontal profile class and incident ischemic stroke by analyzing data from the Atherosclerosis Risk in Communities (ARIC) study.^24^ In the dental ARIC study, 6736 dentate subjects were assessed for periodontal disease status with a total of 299 incident ischemic strokes over the 15-year period. Specifically, individuals with periodontitis presented over twice the risk of cardioembolic and thrombotic stroke compared to those without periodontal issues. This implied that periodontitis may not only promote plaque enlargement, but may also be involved in AS-related thrombosis, which in turn elevates the incidence of thrombotic cerebrovascular events.

The association of periodontitis with unstable AS plaque is also a prerequisite for plaque rupture-induced thrombosis. Brun et al. conducted a cross-sectional study admitted 45 patients for carotid endarterectomy and preoperative periodontal examination. The vulnerability of carotid plaques was assessed based on the volume of intraplaque hemorrhage, indicated by hemoglobin levels released in carotid-conditioned media. The study demonstrated significant inverse correlations between clinical attachment loss (CAL) /serum anti-*P. gingivalis* Immunoglobulin (Ig) A and cytokine inhibiting neutrophils, highlighting their role in plaque stability. There were also significant positive correlations between lipopolysaccharides (LPS) and hemoglobin levels, suggesting periodontitis was significantly associated with carotid plaque vulnerability to rupture.^51^ On the other hand, Bobetsis et al. used gray scale median (GSM) score from doppler ultrasound and immunohistochemistry of CD68 and anti-alpha-actin as metrics for evaluating plaque stability. They found several periodontal clinical parameters, including CAL, pocket probing depth (PPD), and bleeding on probing (BOP), were strongly correlated with GSM, macrophage aggregation, and smooth muscle cells density,^52^ which further reinforces the association between periodontitis and an increased risk of carotid plaque rupture. In accordance with this, by using coronary computed tomography angiography (CTA) and periodontal examination of patients with unstable angina, Rodean et al. found that high periodontal indices was significantly and positively associated with coronary artery calcification scores and a more vulnerable plaque phenotype.^53^ The above evidence indirectly suggests that periodontitis is a risk factor for secondary cerebral or myocardial thrombotic infarction following plaque rupture.

AS-related thrombosis in PAD can manifest as noticeable intermittent claudication, rest pain, gangrene or ulceration in the lower extremity, and in the most severe cases, acute limb ischemia (ALI), amputation and even death. Therefore, the relationship between atherothrombosis and periodontitis can be studied more intuitively by investigating clinical data on PAD compared to cardiac or cerebrovascular diseases. Using the Korean National Health Insurance Service-Health Screening Cohort (NHIS-HEALS) database, Cho et al. compared the incidence of PAD in patients with periodontitis to a matched control group selected from among 514,832 people enrolled in the NHIS-HEALS database. They discovered that the hazard ratio (HR) for PAD in the periodontitis group compared to the matched group was 1.15 (95% confidence interval, 1.07-1.23). This increased occurrence of PAD in patients with periodontitis was concluded despite correction for gender, age, smoking, and hypertension.^54^ Similarly, a meta-analysis of 25 studies identified through a comprehensive systematic review indicated that periodontitis is an independent risk factor for the heightened incidence of PAD.^55^ Nonetheless, there remains a lack of solid evidence-based studies confirming a direct association between periodontitis and AS-related thrombosis. Current evidence has primarily focused on the positive correlation between periodontitis and various indicators that indirectly represent plaque stability. The role of periodontitis or periodontal pathogens in thrombosis after the rupture of an AS plaque remains unknown. This could be attributed to the chronic and continuous nature of AS disease progression, making it challenging to isolate the specific clinical event of thrombosis for individual study.

#### Nonatherosclerosis-related thrombosis

##### Thromboangiitis obliterans (TAO)

TAO, also known as Buerger disease or von Winiwarter-Buerger syndrome, is a chronic, nonatherosclerotic, segmental, obliterative, inflammatory vasculopathy characterized by the presence of a highly cellular arterial thrombus and extensive intimal inflammation.^56^ While the etiology of TAO remains unknown, immune-mediated vascular injury has been proposed as a possible mechanism.^56^ Previous studies have identified smoking as a significant risk factor for TAO,^57,58^ yet there are still some patients with TAO who have never used tobacco. It is worth emphasizing that poor oral hygiene and a high prevalence of periodontitis are common among TAO patients. In 2005, researchers identified causative organisms of oral origin from TAO lesions.^59^ A retrospective study involving 58 patients with TAO reported that periodontitis corresponding to moderate grade in 31% of cases, while severe grade and edentulous patients was revealed in 56% and 13%, respectively.^60^ In addition, compared to healthy individuals, patients with TAO not only had a higher prevalence of periodontitis, but also higher serum levels of antibodies related to periodontal pathogens,^61^ and a positive correlation with the percentage of sites with clinical attachment loss ≥ 4 mm.^62^ Consequently, it has been postulated that TAO may be an infectious disease, with periodontitis being the most probable source of infection in these patients.^63^

##### Atrial fibrillation-related thrombosis

Atrial fibrillation (AF) is a chaotic, rapid (300-500 bpm), and irregular atrial rhythm, which is the result of either electrophysiological abnormalities that underlie impulse generation and/or structural abnormalities of cellular connections that typically facilitate rapid and uniform impulse conduction.^64^ Dislodgment of an attached thrombus due to AF can result in acute embolization, and secondary thrombosis around the emboli makes thrombolysis and reperfusion more difficult. Periodontitis is a risk factor for the occurrence of atrial fibrillation. Chen et al. evaluated 393,745 patients with periodontitis as well as 393 745 non-periodontitis subjects using the 1999-2010 Taiwanese National Health Insurance Research Database and found a higher risk of AF in the periodontitis group compared with the non-periodontitis group after adjusting for potential confounders (HR = 1.31; 95% confidence interval, 1.25-1.36).^26^ Interestingly, the higher risk of AF in the periodontitis group remained significant in all disease subgroups (except hyperthyroidism and sleep apnea). Miyauchi et al. conducted a study on 596 patients with AF who underwent a first session of radiofrequency catheter ablation for recurrence with a mean follow-up period of 17.1 ± 14.5 months and tested the serum IgG antibody titers against *P. gingivalis* (types I-IV). Multivariate Cox proportional analysis demonstrated that high-value serum antibody titer against *P. gingivalis* type IV independently predicted late AF recurrence, providing further evidence of the association between periodontitis and AF.^65^ In addition, Miyauchi et al. categorized left atrial appendage spontaneous echo contrast (LAA-SEC), a thrombosis indicator, into nondense SEC and dense SEC based on echocardiography images and found out that high-value serum antibody titer of *P. gingivalis* type II and type IV was associated with dense SEC, which suggested that *P. gingivalis* may aggravate LAA prothrombotic status and periodontal health or treatment may be necessary for patients with elevated serum *P. gingivalis* type II and type IV antibody level.^66^ Meanwhile, another study also indicated that periodontitis has a strong connection with thrombosis events in patients with AF.^67^ Sen et al., in their analysis involving 5958 individuals, successfully established an association between PD, dental care utilization and incident. They also explored AF as a mediator to PD-stroke association using the dental cohort of the Atherosclerosis Risk in Communities Study.^68^ This indirectly attests to the connection between periodontitis and embolization-induced non-atherosclerotic secondary thrombosis.

##### Arterial dissection-related thrombosis

Secondary thrombosis from arterial dissection can lead to serious ischemic cardiovascular events or increase the risk of arterial dissection rupture, which can lead directly to death.^69^ Delgado et al. reported a case of severe carotid artery dissection caused by periodontal infection of the second inferior molar and cervical cellulitis, suggesting that severe periodontal infection may be a contributing factor in the development of arterial dissection.^70^

Marfan syndrome (MFS) is an autosomal dominant connective tissue disorder caused by missense mutations in fibronectin-1 (FBN-1), a component of extracellular microfibrils. Cardiovascular complications including aortic root dilatation, coarctation, and rupture are the primary contributor to MFS mortality.^71^ Umezawa et al. conducted an 8-month follow-up evaluation of periodontal parameters and cardiac function markers in 3 pre-surgery MFS patients, 4 post-surgery MFS patients, and 7 healthy volunteers. During this period, all patients underwent supportive periodontal therapy (SPT). The study revealed that the BOP and periodontal inflamed surface area (PISA) of MFS preoperative patients were higher at baseline compared with the control group. Even more interestingly, cardiac function indicators, such as left ventricular end-diastolic diameter (LVDd) and ejection fraction (EF) values, were significantly improved in the pre-surgery MFS patient group after SPT.^72^ This study showed that MFS patients exhibit a deteriorated periodontal condition, while periodontal treatment exerts a positive impact on the cardiovascular function of MFS patients. Not only that, both LVDd and EF are important indicators for predicting the occurrence of thrombosis,^73,74^ suggesting a potential relationship between periodontitis or periodontal therapy and thrombosis induced by arterial dissection.

### Periodontitis and venous thromboembolism

Venous thromboembolism (VTE), which encompasses deep venous thrombosis (DVT) and pulmonary embolism (PE), are the most common preventable cause of hospital death and a source of substantial long-term morbidity. A study conducted in 2007 proposed oral bacteria as a risk factor for valvular incompetence in primary varicose veins.^75^ Subsequently, a cross-sectional study involving 197 individuals found that a high prevalence of periodontal disease was detected in patients with thromboembolic disease. Various periodontal parameters, including bleeding index, gingival index, simplified oral hygiene index, clinical attachment level, and probing pocket depth, were found to be significantly higher in VTE disease patients than healthy control group.^76^ The same research team also observed a relationship between the presence of periodontitis and elevated D-dimer levels in another cross-sectional, case‑control study that enrolled 142 patients, which suggested that patients with VTE disease and periodontal disease may face an increased risk of experiencing a new episode of thromboembolism.^77^ Furthermore, results from a prospective cohort study comprising 8 092 participants indicated that the VTE incidence rate of 4.53/1 000 person in periodontal disease compared to 2.79/1 000 person without periodontal disease.^78^ Meanwhile, a state-of-the-art systematic review evaluating and including 15 articles drew firm conclusions about the association between periodontal disease and the incidence of VTE.^79^ Last but not least, although DVT mainly occurs in lower extremity, several researches reported that periodontal bacteria can be involved in venous thrombosis within the whole body. Hiral et al. reported a case of Lemierre syndrome (right internal jugular vein thrombosis, peritonsillar and retropharyngeal abscesses) in a patient with a history of poor oral health, gingivitis and periodontitis, results of 16S rRNA sequencing indicated *Dialister pneumosintes*, a common pathogen in periodontal disease.^80^ Another case report described a patient with cerebral venous thrombosis and a history of periodontal disease. The blood culture revealed an *Eikenella corrodens* infection, which likely originated from the patient’s severe periodontal disease.^81,82^ Following administration of antibiotics and anticoagulant therapy, the patient experienced significant improvement in symptoms.

Septic PE (SPE) is a form of PE characterized by pulmonary embolism and focal lung abscesses resulting from bacteremia.^83^ Periodontal infections have been identified as a potential source of infection in SPE cases. However, isolation of the specific causative pathogen associated with periodontal disease-related SPE has proven to be rare, leading to limited understanding regarding its etiology.^84^ Castro et al. isolated two anaerobic bacteria commonly found in oral flora, *Parvimonas micra* (*P. micra*) and *Provetella oralis* (*P. oralis*), from blood cultures of a patient with SPE who had poor oral hygiene. *P. micra* is a common etiologic pathogen of periodontitis or periodontal abscesses, hence the authors hypothesized that periodontitis was the main cause of SPE in this patient while ruling out other potential sources of infection.^85^ Similarly, Watanabe et al. also reported that *P. micra* could be isolated from blood cultures of a patient with SPE with periodontal abscess.^86^ *Fusobacterium nucleatum* (*F. nucleatum*) serves as an accessory pathogen assisting in the colonization of keystone pathogen *P. gingivalis* in periodontitis.^1^ In addition, *F. nucleatum* is closely related to atherosclerosis^87,88^ and ulcerative colitis,^15^ acting as a microbial bridge mediator for periodontitis-associated systemic diseases. Yamada et al. isolated *F. nucleatum* from blood cultures of a patient with SPE with diabetes mellitus. Oka et al. isolated and cultured *F. nucleatum* from the blood of a patient with tooth extraction induced SPE and aseptic meningitis,^89^ suggesting that bacteremia caused by accessory pathogens in periodontitis also has the opportunity to cause SPE in the background of an inflammatory systemic disease. Accordingly, periodontal therapy exerts a favorable effect on the prognosis of patients with SPE. In 2013, two retrospective studies analyzing 9 and 12 cases, respectively, showed that no symptomatic, radiographic, echographic or laboratory findings indicated infectious lesions other than dental lesions in all the cases. It’s worth noting that nine of the cases had SPE induced by dental infection multiple peripheral nodules, wedge‑shaped peripheral lesions along with feeding‑vessel sign. All patients recovered completely after antimicrobial therapy concomitant with tooth extraction or periodontal care.^90,91^ In conclusion, periodontal infection is a critical source of bacteremia to be investigated. Positive clinical outcomes may be achieved with periodontal therapy in combination with antibiotic therapy in SPE patients.

### Periodontitis and autoimmune-related thrombotic diseases

The relationship between inflammation and thrombosis has been intensively studied, indicating immune system and the coagulation system are functionally interrelated. Inflammation-induced thrombosis is now recognized as a feature of systemic autoimmune diseases such as Behçet’s syndrome and anti-neutrophil cytoplasmic antibody (ANCA)-associated vasculitis (AAV) and antiphospholipid syndrome (APS), especially during the active phase of the disease.^92^ Host immune response plays an significant role in the pathological mechanism in periodontitis, thus suggesting that periodontitis and its associated systemic inflammation may potentially contribute to thrombosis by participating in or aggravating the process of autoimmune-related thrombotic diseases.

#### Behçet’s syndrome

Behçet’s syndrome is classified as a chronic inflammatory disease categorized as one of the systemic vasculitis and was originally described as a triad manifestations: oral and genital aphthosis and non-granulomatous uveitis.^93^ Vascular involvement occurs in up to 50% of patients with Behçet’s syndrome, most commonly superficial and deep vein thrombosis. Venous thrombosis at atypical sites and arterial involvement are also unique features of Behçet’s syndrome.^94^ Recurrent and painful ulcers can interfere with a patient’s oral hygiene and lead to dental plaque accumulation. Celenligil-Nazliel et al. evaluated the periodontal status of 33 patients with Behçet’s syndrome and 15 healthy subjects and determined serum antibody responses to major periodontal pathogens in these patients. They found that significantly higher values for each of the clinical measures were observed in patients with Behçet’s syndrome compared to healthy counterparts. Behçet’s syndrome patients demonstrated elevated antibody levels to *Aggregatibacter actinomycetemcomitans* (*A. actinomycetemcomitans*) Y4 in comparison to the control group.^95^ Akman et al. discovered that periodontal index of treatment needs (CPITN) in 86 Behçet’s syndrome patients was significantly higher than 82 healthy individuals. In addition, clinical severity scores (CSS) of patients with Behçet’s syndrome were positively correlated with CPITN, suggesting that periodontitis may trigger systemic inflammation leading to the development or progression of Behçet’s syndrome.^96^ The same research team further utilized genotyping techniques to screen that carrying the IL-1β-511T allele as well as TNF-α -1031T/C gene polymorphism may be a key risk factor for periodontitis-related Behçet’s syndrome.^97,98^ Some studies have reported that the use of neodymium-doped yttrium aluminium garnet (Nd:YAG) lasers and medications in combination with periodontal therapy has a favorable short-term effect on the reduction of oral ulcers in patients with Behçet’s syndrome.^99,100^ However, there is still insufficient evidence regarding the impact of periodontal therapy on CSS and pro-thrombotic status in Behçet’s syndrome.

#### ANCA-associated vasculitis (AAV)

AAV is recognized as a systemic vasculitis affecting small vessels, accompanied by the presence of ANCAs in the serum. The major antigens targeted by these ANCAs are myeloperoxidase (MPO) and proteinase 3 (PR3).^101^ In PR3-ANCA patients, plasminogen has been described as an autoantigen that interacts with autoantigens directed against complementary PR3, and is capable of preventing its conversion to plasmin and impairing fibrinolysis.^102^ The prevalence of venous thrombosis in AAV ranges between 5.8% and 30%,^103^ while arterial involvement is estimated to occur in 3.1% and 18.7% of all AAV patients.^104^ Novo et al. examined ANCA level in the serum of 30 patients with systemic lupus erythematosus by ELISA and found a significant correlation between ANCA concentrations and periodontitis, suggesting that periodontitis can stimulate an increase in circulating ANCA concentrations in the background of autoimmune diseases.^105^ Esberg et al. examined the salivary microbiota of 85 patients with pre-asymptomatic AAV, 78 patients with established AAV and matched controls using 16 s rDNA sequencing. The team found that patients with pre-asymptomatic AAV had a higher abundance of periodontitis-associated species in their saliva paralleling more signs of periodontitis in established AAV-patients than controls.^106^ However, Viera et al. found no significant correlation between gingivitis and ANCA concentrations when evaluating plasma ANCA and periodontal tissue radiology in 20 patients with acute leukemia as well as 20 healthy controls.^107^ The authors suggested that ANCA might be associated with immune changes specific to leukemia patients, while the impact of gingivitis on their changes appeared minimal. Therefore, the effect of gingivitis or periodontitis on ANCA in patients with systemic immune imbalances remains to be further investigated. In conclusion, the link between ANCA-induced thrombosis and periodontitis-induced elevated ANCA levels requires further investigation.

#### Antiphospholipid syndrome (APS)

APS is a thrombo-inflammatory disorder that complicates up to one-third of systemic lupus erythematosus cases and portends additional organ damage over time.^108^ Primary APS can also manifest in the absence of other systemic autoimmune diseases. The pathogenesis of APS involves circulating antiphospholipid antibodies, which can lead to vascular thrombosis and obstetric complications.^109^ For APS patients, venous thrombosis predominantly affects the deep veins of the lower extremities, while arterial thrombosis primarily targets cerebral arteries.^110,111^ Moreover, APS-related thrombosis may also occur in sites where thrombus do not commonly form in the general population, including the arteries that supply blood to the intestines and the dural venous sinuses of the brain. Meanwhile, APS patients are also at risk for microvascular thrombosis in the skin, eyes, heart, lungs, kidneys or other organs.^112^ Infectious diseases are thought to be potential triggers for APS,^113^ indicating that periodontitis may promote APS.

Anticardiolipin antibodies, also named as anticardiolipin (aCL), are one of the key markers used in laboratory examinations to diagnose APS.^109^ In systemically healthy individuals with periodontitis, approximately 15% to 20% have higher aCL serum levels than 95% of the general population or healthy individuals without periodontitis.^114^ This observation suggested a possible relationship between periodontitis and APS. Additionally, infection with periodontal pathogen *P. gingivalis* can also induce the expression of aCL in mouse serum.^115^ The induction of aCL expression by microorganisms is achieved through the molecular mimicry of β2 glycoprotein I. Apart from *P. gingivalis*, both *A. actinomycetemcomitans* and *Treponema denticola* (*T. denticola*) possess sequences homologous to β2GPI,^62,116^ which elucidates the promotion effect of periodontitis on aCL expression.

Annexin V is an intracellular phospholipid-binding protein that specifically binds to cell membranes of exposed anionic phospholipids, thereby self-assembles into a two-dimensional lattice on the cell surface.^117^ This self-assembly of annexin V serves as a protective “barrier” against fibrin clot formation in various cells such as trophoblasts and endothelial cells.^118^ However, the interaction between serum aCL and annexin V disrupts this protective effect, contributing to one of the key pathogenic mechanisms of APS and related adverse pregnancy outcomes.^119,120^ Schenkein et al. used aCL derived from the serum of periodontitis patients to promote miscarriage in a mouse pregnancy model. Subsequent in vitro experiments demonstrated that the interaction between aCL and annexin V in periodontitis serum is the mechanism of this phenomenon.^121^ Therefore, the high expression of aCL under periodontitis may promote the progression of APS through the annexin V pathway.

## The correlation mechanism between periodontitis and thrombotic diseases

**Fig. 2:**
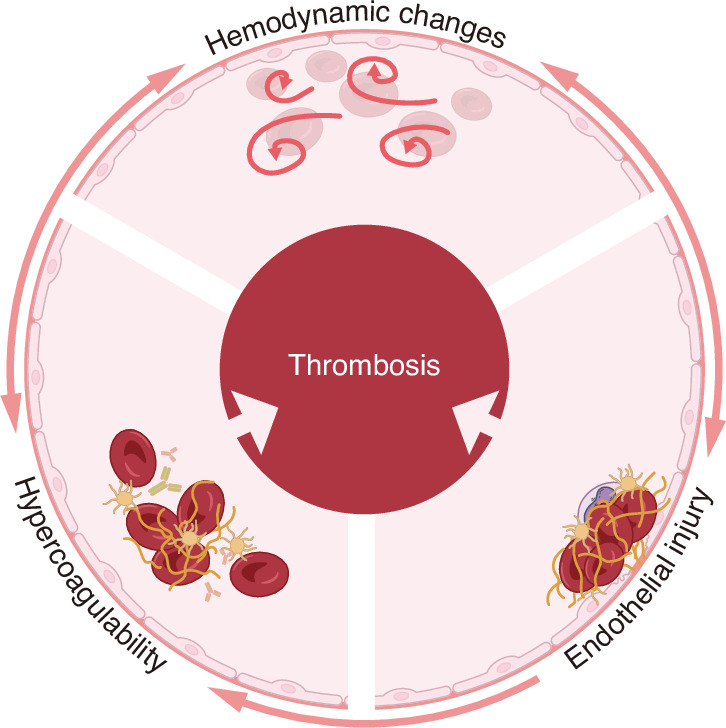
Endothelial injury, hypercoagulability and hemodynamic changes constitute Virchow’s triad. Specifically, endothelial injury and hypercoagulability are the predominant mechanisms underlying periodontitis-related thrombotic diseases, which may indirectly contribute to hemodynamic changes and ultimately culminate in the formation of thrombosis. The illustration was created by C.W

**Fig. 3:**
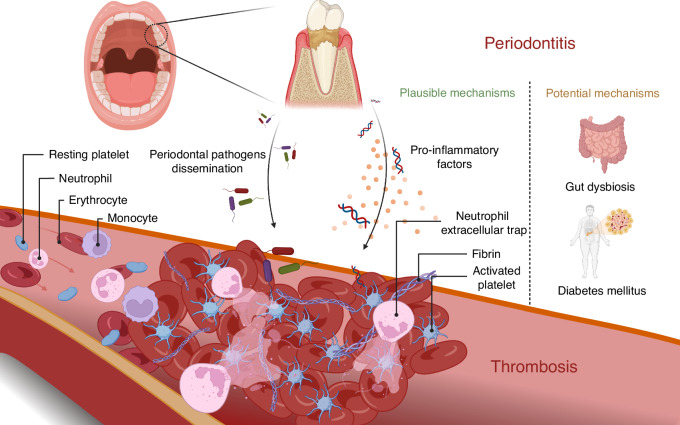
Periodontitis induces systemic inflammation and promotes thrombosis through bacterial dissemination and the release of pro-inflammatory factors into the circulation. Platelet activation and expression of neutrophil extracellular traps are important features in thrombosis with periodontitis. Gut dysbiosis and diabetes mellitus are also potential mechanisms by which periodontitis accelerates thrombosis. The illustration was created by X.Z. using BioRender.com

It is revealed that periodontitis contributes directly to the hypercoagulability and endothelial injury of the Virchow’s triad, while indirectly causing blood flow sluggish and promoting thrombosis formation (Fig. 2). Extensive studies have implicated bacteremia induced by periodontal infection, as well as dysbiosis in systemic immune homeostasis, as underlying mechanisms linking periodontitis to thrombosis disease (Fig. 3).

### Systemic bacterial dissemination

A combination of keystone pathogens represented by *P. gingivalis*, *T. denticola* and *Tannerella forsythia* (*T. forsythia*), *A. actinomycetemcomitans*, along with accessory pathogens such as *F. nucleatum* results in periodontal infections and subsequent periodontium destruction.^1,2^ Apart from being transmitted through the bloodstream, periodontal bacteria may hijack leukocytes or erythrocytes via a C3b–complement receptor 1 interaction, to disseminate from the periodontal pocket to distal vascular system.^1^ These bacteria not only invade endothelial cells, they also promotes the migration of leukocytes that may harbor intracellularly surviving bacteria by up-regulating endothelial cells expressed adhesion molecules and chemokines.^1^ Notably, serum immunoglobulin level against *A. actinomycetemcomitans* was positively correlated with von Willebrand Factor (vWF) concentrations in patients with periodontitis, suggesting an association between periodontal pathogens infection and prothrombotic state.^122^ A study conducted by Range et al. indicated that DNA from periodontal bacteria is identified in 73% of carotid atherosclerotic plaques in patients.^123^ *P. gingivalis* DNA can also be detected in thrombotic material in acute MI patients treated with primary percutaneous coronary intervention (PCI).^124^ Moreover, Joshi et al. conducted a systematic review including 14 researches to investigate the prevalence of periodontal pathogens in coronary artery plaque and/or thrombi from patients with MI.^125^ Meta-analysis indicated that *P. gingivalis* was the most commonly detected microorganism and *A. actinomycetemcomitans* was another major periodontal pathogen detected in the species of MI patients, despite the prevalence of *P. gingivalis* was significantly higher than *A. actinomycetemcomitans*. Apart from *P. gingivalis* and *A. actinomycetemcomitans*, various microorganisms DNA including *Pseudomonas fluorescens*, *Streptococcus* species and *Chlamydia pneumoniae* were also found in the plaque and/or thrombi samples. In addition, oral bacterial DNA had been identified in 13 specimens of arterial thrombus specimens from 14 patients with TAO, whereas none of the control arterial samples was positive for periodontal bacteria.^59^ A case report demonstrated that untreated periodontitis may progress to septicemia and result in septic cavernous sinus thrombosis and PE due to sustained prothrombotic status, ultimately increasing the risk of developing VTE episodes considerably.^126^ A study of bacterial signatures in thrombus aspirates of patients with lower limb arterial and venous thrombosis indicated the majority 80% of bacterial DNA-positive cases contained DNA from the *Streptococcus mitis* group, which is one of the dominant bacteria that constitute the oral biofilm,^127^ suggesting oral bacteria dissemination in the blood circulation is widespread, regardless of the presence or absence of periodontitis.

Platelets are the cytoplasm shed from the cytoplasmic lysis of mature megakaryocytes in the bone marrow. The physiologic function of platelets is in hemostasis and to maintain the integrity of the vascular system. However, excessive activation of platelets can significantly increase the risk of thrombosis. Platelets play an essential role in the pathogenesis of periodontitis itself. Platelet size was significantly reduced in gingival specimens from patients with generalized aggressive periodontitis compared to healthy controls, suggesting consumption of large platelets in periodontal inflammation.^128^ Immunofluorescence staining revealed the interaction between platelets isolated from gingival crevicular fluid (GCF) of periodontitis patients and bacteria as well as neutrophils. This was further confirmed by using *P. gingivalis* and *F. nucleatum* co-cultured with platelets isolated from GCF in vitro,^129^ indicating that periodontal pathogens may be involved in thrombosis formation via the platelet pathway. Several in vitro studies corroborate the effect of periodontal bacteria on platelet function. Specifically, *P. gingivalis*, a representative periodontal bacteria, can promote platelet activation and aggregation through toll-like receptor (TLR) 2 and TLR4 signaling pathways,^130–133^ stimulating platelets to over-express P-selectin,^134^ CD40L^131^ and under-express basal and prostaglandin E1 induced vasodilator-stimulated phosphoprotein-phosphorylation, thereby transmitting adverse signals to other cells in the vascular system,^135^ Additionally, human aortic endothelial and smooth muscle cells increase tissue factor production and exhibit low expression of tissue factor pathway inhibitors under *P. gingivalis* stimulation.^136,137^ Taken together with the previous evidence, these illustrated that periodontal bacterial infections place the vascular system in a prothrombotic state.

A pilot study showed positive correlations between platelet indices (mean platelet volume and platelet distribution width) and PISA in coronary artery disease patients, implicating periodontal pathogens in the development of cardiovascular disease through activation of platelets.^138^ Flow cytometry analysis was performed to quantify activation of platelets in whole blood from patients with periodontitis and controls, with and without stimulation by oral bacteria, finding that in response to several species of oral bacteria, including *P. gingivalis* and *A. actinomycetemcomitans*, platelets from periodontitis patients showed over-expressed P-selectin and increased formation of platelet-monocyte complexes compared with controls.^139^ This reinforces the argument that the dissemination of periodontal bacteria in the blood circulation escalates the risk of thrombosis.

The correlation between periodontal pathogens and thrombosis has been demonstrated in several studies utilizing rat models. By employing a jugular vein infusion pump to continuously infuse *P. gingivalis* into rats for a duration of 2 weeks, researchers observed thrombosis in the iliac, superficial, and infrapopliteal arteries as well as the identification of *P. gingivalis* DNA in the specimens of these blood clots.^140^ *P. gingivalis* could also induce platelet aggregation in rats and P-selectin (CD62P) overexpressing under collagen stimulation in vivo and in vitro.^141^ In another study in vivo, besides replicating the phenomenon that periodontitis promotes platelet aggregation in rats, down-regulation of phosphorylated endothelial nitric oxide synthases (peNOS) expression in the vasculature was observed, hinting that the periodontitis-related platelet phenotype is associated with increased vascular reactivity.^142^ Iwai et al. injected solution containing *P. gingivalis* into rats’ blood vessels, acute inflammation reaction occurred at day 7 and it transformed to chronic fibrosis with macrophages or plasma cells infiltration at day 28. CD3 (Pan T-cells), CD79a (Pan B cells in the rats), and IgG were observed during the process of arterial thrombosis healing.^143^ Iwai et al. further investigated the pathological and immunological difference of thrombosis induced by different periodontal pathogens. Intriguingly, the immunophenotypes of arterial thrombosis induced by distinct periodontal bacteria were different, as reflected by stronger disruption of internal elastic lamina in *A. actinomycetemcomitans*, an increased presence of CD3 (Pan T cells) in *Prevotella intermedia* and a higher abundance of CD79a (Pan B cells) in *P. gingivalis*, proposing a new concept of a possible mechanism of vascular diseases, in which the work of lipopolysaccharides and TLRs is emphasized.^144^ However, current researches did not clearly identify which periodontal pathogen plays a major or decisive role in the pathophysiology of thrombosis.

### Systemic inflammation

Chronic uncontrolled periodontal infection and destruction can subconsciously contribute to the systemic inflammatory burden, hence increase the risk of CVD. A cross-sectional study involving 55 participants revealed that periodontitis is associated with brachial artery endothelial dysfunction and systemic inflammation.^145^ In periodontitis, locally produced pro-inflammatory cytokines, such as tumor necrosis factor-α (TNF-α), interleukin-1β (IL-1β) and IL-6, can enter the systemic circulation and trigger an acute-phase response in the liver characterized by increased levels of C-reactive protein (CRP) and fibrinogen.^1^ In turn, DAMPs released from periodontal tissue destruction can enter the bloodstream and bind to pattern recognition receptors (PRRs) on histiocytes or immune cells in organs, inducing intrinsic immune homeostasis and accentuating vascular inflammation.^16^

Neutrophil extracellular traps (NETs) exert an imperative function in the development of thrombosis.^146^ NETs were present in samples from patients with both arterial and venous thrombosis, indicating their significance.^147,148^ Platelet-neutrophil interaction is responsible for the prothrombotic effects of NETs. Platelets bind to P-selectin glycoprotein ligand-1 (PSGL1) on neutrophils via P-selectin promotes NETosis.^149^ Meanwhile, platelet-derived DAMP high mobility group protein B1 (HMGB1) amplifies NETosis through the receptor for advanced glycation end products (RAGE) in vitro and in vivo.^150,151^ As mentioned previously, periodontal bacteria cause platelet function to hyperactivate, and a number of studies have demonstrated that periodontitis can elevate both local and circulating levels of NETs^152–154^ and HMGB1,^155,156^ implying that platelet-neutrophil interactions may be a potential immune mechanism underpinning the association of periodontitis with thrombosis. However, the relationship between periodontitis-driven NETs and thrombosis had been insufficiently investigated. Moreover, it is worth noting that the histone composition and extracellular DNA sequences in periodontitis NETs may exhibit variations compared to other systemic inflammatory conditions, following the fact that periodontal bacteria species are rather specific.

**Fig. 4:**
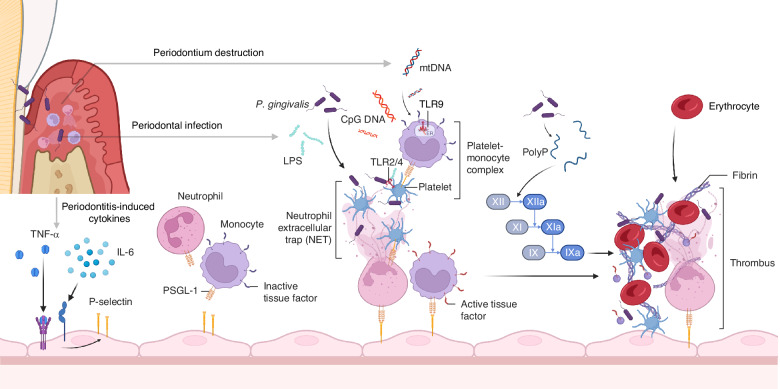
Periodontal infections can lead to bacteremia. Bacteria (eg., *P. gingivalis*) and their metabolites (e.g., LPS) can activate platelets via TLR2/4. Pro-inflammatory cytokines triggered by periodontitis (e.g., TNF-α and IL-6) promote high endothelial cell expression of P-selectin, which is responsible for neutrophil and monocyte recruitment and activation. Neutrophil extracellular traps (NETs) produced by neutrophil activation bind to platelets and accelerate blood cell aggregation. The mtDNA released from periodontal tissue destruction and the CpG DNA released from periodontal bacterial death can activate immune cells through the intracellular nucleic acid receptor TLR9 and accelerate the formation of platelet monocyte complexes. Meanwhile, Poly P produced by periodontal pathogen activates collagen fiber synthesis and participates in thrombus formation by triggering the factor XII-XI-IX cascade signaling mechanism. The illustration was created by X.Z. using BioRender.com

In spite of the extracellular DNA released from NETs, naturally occurring polyphosphates (polyP) such as cell-free nucleic acid (cfNA) and inorganic polyP mediate systemic inflammation as well as procoagulant mechanisms could also be prothrombotic mediators in periodontitis.^157^ It is recognized that cfNA activates coagulation through activation of factors XII, XI and factor VII-activating protease in vitro and in vivo.^158,159^ PolyP released from platelets dense granules is involved in fibrin formation through activation of the factor XII-XI-IX pathway, making the fibrin structure resistant to lysis.^160^ In addition to involvement in coagulation, specific fragments of cfNA, such as CpG DNA and mitochondrial DNA (mtDNA), are direct ligands for the activation of platelet or leukocyte intracellular TLR9, which activates the downstream Myd88-NF-κB signaling pathway to promote the secretion of pro-inflammatory factors by immune cells as well as enhance platelet hyperreactivity, modulating thrombogenesis.^161,162^ Plasma levels of cfNA were significantly elevated in both arterial and venous thrombosis patients compared to the control subjects.^163,164^ Recent studies have demonstrated that periodontitis can elevate local and serum or plasma concentrations of cfNA in patients and this phenomenon was reproduced in ligature-induced periodontitis in mice.^165,166^ Moreover, nucleic acid sensors, represented by TLR9, play a pivotal role in the pathogenesis of periodontitis.^166,167^ The mtDNA derived from the destruction of periodontal tissues and the CpG DNA released by lysis of periodontal bacteria may be responsible for the elevated cfNA in the circulation.^168,169^ Interestingly, in a recent study in vitro, researchers used confocal microscopy which observed the behavior of *P. gingivalis* to initiate coagulation by secreting inorganic polyP.^170^ In summary, inflammatory mediators represented by polyP may be a potential mechanism for the prothrombotic effects of periodontitis (Fig. 4).

Periodontitis may contribute to the progression of systemic inflammatory diseases by causing disturbances in the gut microbiota.^171^ Periodontal pathogens can impair the diversity of the gut microbiota and the integrity of the intestinal mucosal barrier via the oral-gut axis.^1^ Researchers have recently discovered in ApoE^−/−^ mice, a classical model of atherosclerosis, that periodontitis promotes atherosclerosis progression through oral-gut axis mechanisms.^172–174^ Specifically, *P. gingivalis* induces chronic inflammation in the liver by altering the gut microbiota and promotes increased expression of trimethylamine N-oxide (TMAO, a pro-atherosclerotic factor) catalase in the liver.^173^ However, although non-surgical periodontal treatment reversed the effects of ligature-induced periodontitis on gut microbiota dysbiosis in ApoE^−/−^ mice, it had no significant effect on blood lipid profile.^174^ Furthermore, Zhu et al. have suggested that the presence of gut microbes may directly contribute to platelet hyperreactivity, thereby increasing the risk of thrombotic events through the production of TMAO.^175^ It has also been demonstrated that plasma or serum levels of TMAO can serve as a predictive factor for the formation of thrombosis formation.^175,176^ Nemet et al. demonstrated that phenylacetylglutamine (PAGln), a metabolite derived from the gut microbiota, was associated with an increased risk of cardiovascular disease (CVD) and major adverse cardiovascular events (MACE).^177^ Specifically, PAGln was found to enhance platelet activation-related phenotypes and thrombosis potential both in vivo and in vitro. Another study implicated that 2-methylbutyrylcarnitine (2MBC), a branched-chain acylcarnitine produced in a gut-microbiota-dependent manner, can bind to integrin α2β1 in platelets, leading to activation of cytosolic phospholipase A2 (cPLA2) and increased platelet reactivity. This connection links gut microbiota dysbiosis to heightened thrombotic risk. The pro-thrombotic effects of 2MBC can be reversed through genetic depletion or targeted inhibition of α2β1.^178^ Nevertheless, it is worth noted that current evidence regarding the role of oral-gut axis in the mechanism of periodontitis impacts on thrombosis are mostly indirect. There is a limited number of studies that directly investigate the mechanisms by which periodontitis affects crucial thrombosis processes, such as platelet activation and aggregation, through the oral-gut axis. Further in vivo and in vitro studies are needed.

DM is an important risk factor for thrombotic diseases.^179^ There is a bidirectional interaction between periodontitis and DM.^180–182^ Metabolic imbalances triggered by hyperglycaemia could induce systemic inflammation which may promote thrombosis.^183,184^ Several studies have observed platelet hyperactivity in patients with DM.^185–187^ However, it has also been shown that DM has no significant effect on the platelet transcriptome (aggregation and thrombosis-related genes).^188–190^ A single-cell sequencing study of platelets from periodontitis patients and periodontitis-combined DM patients found increased platelet activation in both groups compared to the healthy subjects, yet the expression of thrombosis-related genes, such as CFL1, PFN1, S100A8, and CTSS, as well as the scores of pathways related to “platelet activation, signaling, and aggregation” were lower in periodontitis-combined DM patients compared to periodontitis patients.^191^ In addition, the expression of platelet major receptors such as α_IIb_β_3_, GPIb-IX-V complex, α_2_β_1_, SELPCLEC1B, and TBXA2R was also down-regulated in periodontitis-combined DM patients compared with periodontitis patients.^191^ The findings implied that periodontitis facilitates blood coagulation through platelet dysregulation. Conversely, comorbidities associated with periodontitis and DM compromise the integrity of platelet components, thereby hindering their regular functions. Hence, the role of DM between periodontitis and thrombosis remains to be investigated.

## Clinical implication

### Current therapeutic options and the absence of oral health management in the prevention and treatment of thrombotic diseases

Currently, lifestyle intervention and oral medications are the main options of preventing and treating arterial thrombotic diseases except for surgical treatment, which are recommended by the clinical guidelines of the European Society for Vascular Surgery (ESVS) and American Society of Hematology (ASH). Lifestyle and health behavior intervention, including smoking cessation and aerobic physical activity, is recommended by ESVS guidelines to prevent the risk of disease development and major cardiovascular complications.^192^ In addition, single or dual anti-platelet medications (aspirin and clopidogrel) plus anticoagulation therapy (warfarin or direct oral anticoagulants) are widely recommended by current guidelines in various diseases to prevent thrombosis formation and recurrence.^193^ As for VTE patients, pharmacological thromboprophylaxis and anticoagulation therapy are recommended by ESVS and ASH to reduce the incidence of VTE and its treatment, especially in patients with high risk such as malignancy.^193,194^ Meanwhile, mechanical thromboprophylaxis may also be benefit for the prevention and prognosis of DVT, which is also recommended by ASH group.^194–196^ As we have mentioned before, periodontitis is associated with higher risk of various thrombotic diseases, moreover, previous studies revealed that periodontitis is associated with the development, severity and prognosis of thrombotic diseases (discussed in the following section). In addition, Molania et al. utilized OHIP-14 questionnaire to investigate the oral health-related quality of life (OHRQoL) in patients with CVD and found out that CVD patients had poor OHRQoL, which suggested the imperative of delivering tailored oral disease prevention or treatment interventions to individuals with CVD.^197^ However, there is a significant absence of oral health management in the current clinical guidelines or practice for thrombotic diseases and the number of related literatures is limited, which requires more high-quality studies to determine the role of oral health surveillance or therapy.

### Periodontitis was associated with worse clinical outcome in thrombosis events

Many studies suggest that periodontitis is not only strongly associated with the development of thrombotic diseases, but also has a long-term impact on the prognosis of patients with thrombotic diseases, although these adverse events are not always thrombosis-related.

Acute coronary syndrome (ACS) is a typical thrombotic disease with high lethality. Renvert et al. found a high prevalence of ACS in patients with periodontitis, and a three-year follow-up found that alveolar bone defects caused by periodontitis were associated with distant recurrent practice in patients with ACS, which seriously affected patient prognosis.^198^ Dorn et al. followed 884 patients with heart attacks and found that CAL was associated with recurrent fatal/non-fatal cardiovascular-related events.^199^ Reichert et al. found a trend between severe periodontitis and new cardiovascular events after coronary artery bypass grafting (CABG), although there was no statistically significant difference.^200^ These may be caused by the lack of comparison between patients with mild-to-moderate periodontitis and healthy individuals in the study, as well as the limited follow-up time and number of subjects. Another study found that skin autofluorescence (sAF), a potential measure of periodontitis severity, showed an association with adverse events after CABG.^201,202^ Fukushima et al. found that the severity of periodontitis represented by community periodontal index (CPI) was associated with an increased risk of MACE after percutaneous coronary intervention (PCI) in patients treated with drug-eluting stents (DES) for de novo coronary lesions.^203^ Another study found that 64.1% of patients undergoing PCI had severe periodontitis, and also found that severe periodontitis was associated with the risk of post-PCI in stent re-stenosis (ISR).^204^ Other study assessed periodontal inflammation using the modified total dental index (TDI). The modified TDI was found to be significantly associated with the development of ISR after ACS.^205^ All of this evidence supports that poor periodontal condition is detrimental to postoperative recovery in patients with thrombosis.

As for VTE patients, existing studies have found that patients with periodontitis have a 60% higher overall risk of VTE than healthy people, and the risk of VTE treatment is increased by about 90%, and periodontitis is associated with a higher risk of VTE recurrence.^77^ D-dimer is a biomarker commonly used in clinics to screen and predict prognosis for DVT and PE,^206^ and it has been found that periodontitis can cause elevated levels of D-dimer in the blood, and the magnitude of the elevation increases with the severity of periodontitis. It was found that periodontitis caused an increase in D-dimer levels in the blood, with the increase increasing with the severity of periodontitis, and a significant decrease in blood D-dimer levels was observed after 4 weeks of periodontal treatment.^207^ These results were further confirmed in a study by Sánchez-Siles et al., which included 142 patients with VTE without an obvious cause (76 with DVT, 37 with PE and 29 with both DVT and PE; 71 with periodontal disease and 71 periodontal healthy patients included). The study found that VTE patients with periodontitis had 56% higher D-dimer levels than those without periodontitis, and there was a direct relationship between high D-dimer levels and PE severity.^77^ Another study found that treating severe periodontitis lowered blood levels of fibrinogen and platelets.^208^ In summary, there is a direct relationship between periodontitis and VTE, and it may be necessary to clarify periodontal health status in people at high risk of VTE, such as patients with malignancy and postoperative patients with prolonged/traumatic surgery, and to closely test blood D-dimer levels while giving physical prophylaxis or prophylactic anticoagulation to avoid fatal VTE events.

Overall, the combination of periodontitis may result in higher risk and worse clinical outcome for thrombotic diseases patients. More attention, including early intervention and regular follow-up, should be paid to the prognosis and complications of these patients in clinical treatment.

### Periodontal therapy improves thrombosis markers and has potential benefit for prognosis of patients with thrombotic diseases

There have been some initial basic or clinical studies attempting to intervene in the link between periodontitis and thrombosis markers and periodontal therapy is found to reduce the level of inflammatory thrombosis markers. Serum-hypersensitive C-reactive protein (hs-CRP) can be used as a marker in CVD.^209^ Serum hs-CRP is produced in the SMCs of the coronary artery due to endothelial dysfunction^210^ while healthy periodontium do not produce hs-CRP.^211^ Previous studies have found that hs-CRP levels are elevated in patients with chronic or aggressive periodontitis, and the concentration of serum hs-CRP increases and then decreases after periodontal therapy.^212^ Another multicenter RCT study found that periodontal care reduced hs-CRP levels, which could be considered as a secondary prevention for CVD.^213^ Flow-mediated dilatation (FMD), a measure of endothelial function, was significantly elevated in patients in the treatment group compared to the control group after two months of intensive periodontal treatment, and was also significantly correlated with periodontal health.^214^ Furthermore, periodontal treatment improved endothelial function in patients with acute ST-segment elevation heart infarction.^215^ Another randomized controlled trial (RCT) study also found that periodontal therapy improved postoperative FMD in patients with MI, while both studies confirmed that periodontal treatment was very safe and did not produce any side effects in patients.^216^ Bokhari et al. found that non-surgical periodontal therapy lowered CAD risk markers, such as CRP, white blood cell, and fibrinogen levels in patients with imaging-confirmed coronary artery disease.^217^ It suggests that in these patients, periodontal therapy may improve endothelial cell function without increasing the corresponding risk, thus provide potential positive impact on the prognosis of patients.

Platelet hyperactivation is one of the major mechanisms of thrombosis. Local trauma and transient bacteremia that may be introduced by periodontal therapy do not lead to excessive activation of platelets systemically.^218^ In contrast, prompt therapy for periodontitis limits platelet hyper-reactivity, as evidenced by reduced expression of, P-selectin, CD40L, binding of PAC-1, CD63, and platelet leukocyte complexes in patients.^43,219^ In addition, periodontal scaling and root planing reduce serum aCL concentrations, suggesting that periodontal therapy may be valuable in preventing thrombosis in patients with autoimmune diseases.^220^ However, the results of a RCT study showed that periodontal treatment does not improve the primary outcome of vascular inflammation significantly in patients with PAD combined with periodontitis determined by ^18^F-FDG PET/CT values, possibly due to the observation period of only 3 months.^221^ Nonetheless, taking the above studies together, periodontal therapy remains potentially beneficial for platelet function.

Moreover, current researches suggest that periodontal therapy may reduce the incidence of thrombotic diseases and slow down the disease progression. Gabrione et al. included 34 RCT studies and reviews to test the effects of different periodontal therapies for patients with CVD, and concluded that periodontal therapy can prevent the risk of CVD.^222^ A cohort study that included 2415,963 participants’ data retrieved from the National Health Insurance Database of the Korean National Health Insurance Service suggested that improved oral hygiene, including tooth brushing and periodontal scaling, is associated with a lower risk of venous thromboembolism.^223^ Meanwhile, a population-based cross-sectional cohort study included data from 3271 participants aged between 45 and 74 years enrolled in the Hamburg City Health Study have found that presence of severe periodontitis was independently associated with the development of PAD.^224^ In addition, by using multivariable regression methods, Aarabi et al. included 70,944 hospitalized patients with a diagnosis of symptomatic PAD and found that patients with PAD may have a reduced risk associated with progression to chronic limb-threatening ischemia (CLTI) after receiving periodontal therapy.^225^

In conclusion, periodontal treatment may not only have a positive effect on the expression level of thrombosis markers, but also disease incidence and development, hence to be more favorable for their overall prognosis.

### Future prospective

As mentioned in the previous section, certain periodontal parameters have been identified as potential risk factors or prognostic predictors for diseases such as CVD/PAD/VTE, including CPI, CAL and modified TDI. In addition, periodontal therapy has been found to be effective to reduce thrombosis indicators level and have potential benefit of prognosis of thrombotic diseases patients. However, further investigation is needed to establish a predictive model and possible grading system for periodontally related thrombotic diseases by identifying additional risk factors, prognostic predictors, and biomarkers. Although existing studies have initially demonstrated the underlying impact of periodontitis in promoting thrombosis diseases and influencing their prognosis, the periodontal conditions of patients with thrombotic diseases remains unknown because these periodontal related-index are not routinely observed and documented during the treatment. Taken all these together, the authors believe that more high quality, large samples, multicenter randomized controlled trials are required. In addition, our understanding of the mechanisms underlying how periodontitis promotes thrombosis is currently limited and require more comprehensive experiments in basic medical sciences to identify and validate specific signaling pathways or bridging molecules both in vivo and in vitro. Meanwhile, the potential role of periodontal treatment in the multidisciplinary diagnosis and management of thrombotic diseases necessitate further investigation.

In this perspective review, the authors would like to raise several questions and research directions.

1. Can the periodontal health condition serve as a potential classification method for thrombotic diseases?
2. Does periodontal therapy effectively reduce the incidence of thrombosis or disease progression in high-risk patients?
3. Is routine periodontal care essential for thrombotic diseases patients who require surgery intervention?
4. To what extent does periodontal treatment impact the prognosis of patients with thrombotic diseases?
5. Through which key signal pathway or biomarkers periodontitis poses its influence on thrombotic diseases and is it possible to develop targeted therapeutic options?

**Fig. 5:**
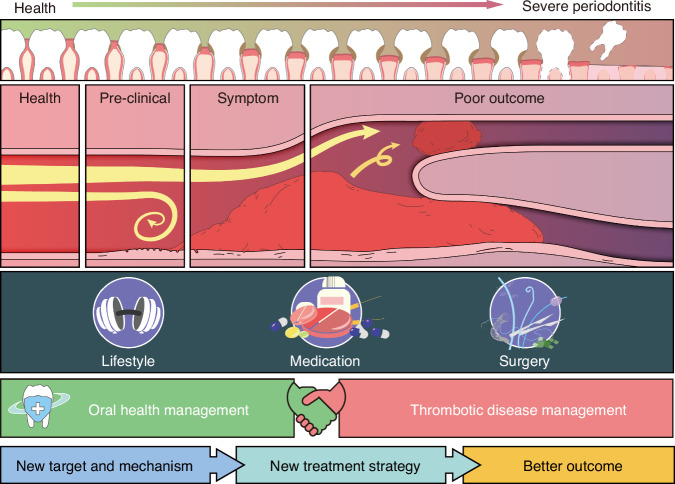
Future perspective regarding the potential role of oral health management in the treatment of thrombotic diseases. Numerous studies have consistently demonstrated the impact of periodontitis on thrombotic diseases across various stages, ranging from pre-clinical to post-operative. Moreover, it has been observed that periodontal treatment positively influences the prognosis of patients with thrombotic diseases. However, current therapeutic approaches for thrombotic conditions primarily revolve around lifestyle modifications, medications, and surgical interventions. The significance of oral health management in preventing and treating thrombotic diseases is currently underappreciated, while the underlying key molecules and pathways through which periodontitis exacerbates these conditions remain elusive. It is essential to integrate oral health surveillance and management into the overall treatment plan for individuals with thrombotic diseases. Additionally, further exploration of the intricate mechanisms by which periodontitis impacts thrombosis can pave the way for potential targeted therapeutic strategies aimed at improving patient outcomes. The illustration was created by C.W

Nevertheless, the exploration and transformation of thrombotic diseases treatment extend beyond conventional medication and surgical approaches, emphasizing the urgent need to elucidate the potential role and effect of periodontal therapy on thrombosis as well as the underlying mechanism linking periodontitis and thrombotic diseases, thereby shaping the future landscape of multidisciplinary treatment (MDT) for such conditions and providing better outcome and new therapeutic options for these patients (Fig. 5).

## Summary

The high prevalence of periodontitis and the potential systemic harm it poses make the management of oral health in people and patients at risk for cardiovascular disease extremely important. This review examined the potential mechanisms by which periodontitis promotes thrombosis through bacteria as well as inflammation in light of the clinical association between periodontitis and various types of thrombotic diseases. Periodontitis impairs prognosis in patients with thrombotic disease. Hence, we present a perspective that periodontal health surveillance should be considered for people at risk of thrombosis and periodontal interventions may be potentially beneficial for prevention and treatment of patient with thrombotic diseases. Unlike atherosclerosis, the correlation mechanism between periodontitis and thrombosis remains to be elucidated, and we are expecting the discovery of more bridge-mediated therapeutic targets and intervention modalities in the future.
